# Association between socioeconomic position and the prevalence of type 2 diabetes in Ghanaians in different geographic locations: the RODAM study

**DOI:** 10.1136/jech-2016-208322

**Published:** 2017-03-27

**Authors:** Juliet Addo, Charles Agyemang, Ama de-Graft Aikins, Erik Beune, Matthias B Schulze, Ina Danquah, Cecilia Galbete, Mary Nicolaou, Karlijn Meeks, Kerstin Klipstein-Grobusch, Silver Bahendaka, Frank P Mockenhaupt, Ellis Owusu-Dabo, Anton Kunst, Karien Stronks, Liam Smeeth

**Affiliations:** 1 Department of Non-communicable Disease Epidemiology, London School of Hygiene and Tropical Medicine, London, UK; 2 Department of Public Health, Academic Medical Center, University of Amsterdam, Amsterdam, The Netherlands; 3 Regional Institute for Population Studies, University of Ghana, Legon, Accra, Ghana; 4 Department of Molecular Epidemiology, German Institute of Human Nutrition Potsdam-Rehbruecke, Nuthetal, Germany; 5 Julius Global Health, Julius Center for Health Sciences and Primary Care, University Medical Center Utrecht, Utrecht, The Netherlands; 6 Division of Epidemiology & Biostatistics, School of Public Health, Faculty of Health Sciences, University of the Witwatersrand, Johannesburg, South Africa; 7 International Diabetes Federation, Kampala, Uganda; 8 Institute of Tropical Medicine and International Health, Charité—University Medicine Berlin, Berlin, Germany; 9 Kumasi Centre for Collaborative Research in Tropical Medicine, Kwame Nkrumah University of Science and Technology, Kumasi, Ghana

**Keywords:** Epidemiology of diabetes, SOCIO-ECONOMIC, INEQUALITIES, MIGRATION

## Abstract

**Background:**

The prevalence of diabetes has been shown to be socially patterned but the direction of the association in low-income countries and among migrant populations in Europe has varied in the literature. This study examined the association between socioeconomic position (SEP) and diabetes in Ghanaians in Europe and in Ghana.

**Methods:**

Data were derived from the multicentre Research on Obesity and Diabetes among African Migrants (RODAM) study of Ghanaian adults aged 25–70 years residing in Europe (Amsterdam, Berlin and London) and in urban and rural Ghana. Educational attainment (elementary, secondary or higher) and occupational class (low or high) were used as indicators of SEP. Age-standardised prevalence of diabetes and prevalence ratios were evaluated separately for men and women of different SEP in Ghana and Europe.

**Results:**

A total of 5290 participants were included in the analyses. The prevalence of diabetes decreased with increasing level of education in Ghanaian men and women in Europe and in men in urban Ghana, whereas diabetes prevalence increased with increasing level of education in men and women in rural Ghana. The association between occupational class and the prevalence of diabetes followed a less consistent pattern in men and women in the different locations.

**Conclusions:**

The association of diabetes and SEP differed in rural Ghana compared with urban settings in Ghana and Europe and comparing men and women, highlighting the complex interaction of SEP and the development of diabetes. These findings have important implications for diabetes prevention strategies in Ghanaians in different locations.

## Introduction

Socioeconomic disparities have been reported in the incidence and prevalence of type 2 diabetes (diabetes) in previous studies.^1^ An inverse association has been consistently reported in the association between socioeconomic position (SEP) and the prevalence of diabetes in studies from high-income countries irrespective of the indicator of SEP used.^2–4^ The association between SEP and diabetes in low-income and middle- income countries and in migrant populations has, however, not been consistent over time and in different geographic locations.^5–7^ Gender differences have also been reported in the patterns of the association between SEP and diabetes.^2^ ^8^ Several factors are thought to be implicated in the socioeconomic differences in diabetes prevalence including differences in access to healthcare, differences in obesity prevalence, health-related behaviours and in the availability or affordability of foods and activities supporting a healthy lifestyle.^9^ ^10^ Variations in diabetes in different population groups may be associated with societal and environmental changes associated with urbanisation or migration to western environments.^11^ The limited data available on the association between SEP and cardiovascular disease (CVD) and its risk factors such as diabetes in migrant populations in Europe have not always been consistent with the European pattern of inequalities.^12–14^ Research examining the association of SEP and diabetes in Africans is scarce, yet diabetes has been shown to exert a significant burden in subSaharan Africa (SSA) and in Africans in Europe in whom prevalence similar to the host populations have been reported.^15–18^ Considering the different patterns reported in the association between SEP and CVD risk factors including diabetes in some studies from low-income and middle-income countries and among migrant populations, and the paucity of data in Africans, examining the patterns of socioeconomic inequalities in Africans in different geographic locations is essential to inform prevention strategies. The aim of this study was to examine the association between diabetes and measures of SEP in a relatively homogenous population of Africans (Ghanaians) residing in Europe compared with their non- migrated counterparts living in rural and urban Ghana, explore the differences in men and women in various locations and examine the extent to which mediating factors such as obesity and physical activity explain any observed patterns.

## Materials and methods

### Study population

Data for this study were derived from the Research on Obesity and Diabetes among African Migrants (RODAM) study. The aims and design of the RODAM study have been described previously.^19^ In brief, RODAM is a multicentre cross-sectional study of Ghanaians residing in rural and urban Ghana (Ashanti region of Ghana), the Netherlands (Amsterdam), the UK (London) and Germany (Berlin) aimed at understanding the reasons for the high prevalence of diabetes and obesity among African populations in Europe. The study was conducted between 2012 and 2015. Owing to differences in available data registration systems, different recruitment strategies aimed at obtaining representative samples of Ghanaians in the various sites were employed across locations. In Ghana, participants were randomly drawn from the list of 30 census enumeration areas in the Ashanti region. Participants in Amsterdam were randomly drawn from the Amsterdam Municipal Health register which contains data on the country of birth of residents and their parents. Ghanaian organisations including churches as well as social and community groups served as the sampling frame in London and Berlin. The participation rate was 76% in rural Ghana and 74% in urban Ghana. In London, 75% of individuals registered in the various Ghanaian organisations and invited to participate took part and the figure was 68% in Berlin. In Amsterdam, a response was received from 67% of those invited, and 53% of these participated in the study.

All recruited participants completed a structured questionnaire and a physical examination. Fasting venous blood and urine samples were collected, processed and temporarily stored at −80°C at the local research centres' laboratories according to standard operational procedures and transported to one site (Berlin) for biochemical analyses. Weight was measured twice without shoes and in light clothing to the nearest 0.1 kg using a portable electronic scale (SECA 877). Height was measured twice with a portable stadiometre (SECA 217) to the nearest 0.1 cm.

Ethical approvals were obtained from the respective ethics committees and review boards in the different locations.

### Diabetes

Diabetes was defined as fasting plasma glucose ≥7.0 mmol/L, treatment for diabetes or self-reported diabetes.^20^

### Socioeconomic indicators

SEP was measured by education and occupation. Educational status based on the self-reported highest qualification attained by the participants was classified into: ‘elementary’ (no, primary or lower secondary education), ‘secondary’ (upper secondary and postsecondary non-tertiary education) and ‘higher’ (tertiary education). Occupational classes were coded according to the International Standard Classification of Occupations scheme (ISCO-08) classified as ‘high’ (professionals, managers, clerical support staff, higher grade routine non-manual employees service and sales-related occupations) and ‘low’ (craft and related trades workers, elementary occupations and farmers).^21^

### Assessment of covariates

Other covariates considered were age in categories (25–34, 35–44, 45–54, 55–64 and 65+) and sex. Body mass index (BMI) was classified as ‘underweight’ <18.5 kg/m^2^; ‘normal weight’ 18.5–24.9 kg/m^2^; ‘overweight’ 25.0–29.9 kg/m^2^; and ‘obesity’≥30 kg/m^2^.^22^

The level of physical activity was assessed from the frequency, duration and intensity of different types of major activities in the domains of work-related physical activity, active commuting (walking and cycling) and leisure time physical activity using the Global Physical Activity Questionnaire developed by the WHO.^23^ Based on metabolic equivalents of task (MET) determined per week using the compendium of Ainsworth,^24^ the physical activity level of participants was classified as ‘low’ or ‘high’. Dietary intake was assessed with a standardised Ghana Food Propensity Questionnaire (Ghana-FPQ) that queried the frequency of intake of food groups at predefined portion sizes in the preceding 12 months. The Ghana-FPQ covered 134 food items based on the European Food Propensity Questionnaire in addition to Ghanaian foods.^25^ ^26^ Food intake frequencies were combined with standard portion sizes to estimate the usual daily total energy intake in kcal/day and were classified as <2000, 2000–3500 and >3500.

### Statistical analysis

All statistical analyses were performed using STATA V.14 (Stata Corp, College Station, Texas). Age-standardised prevalence of diabetes was calculated by the direct method with the standard being the age distribution of the total study population. The associations between the SEP measures and the prevalence of diabetes were assessed using Poisson regression with robust SEs, adjusted for age. Possible mediators (BMI, physical activity and daily energy intake) were also included in the models. Analyses were performed separately for men and women. There was no significant interaction between European sites in the association between SEP measures and the prevalence of diabetes and therefore data from Europe were combined in the regression analyses. A total of 5290 participants aged between 25 and 70 years were included in the analyses. There was no missing data for diabetes. The associations between missing data for the key explanatory variables of interest (education n=391 and occupational class n=1236) and diabetes were investigated by site, using χ^2^ tests. For the regression models, a complete case analysis approach was used. To maximise amount of data available, all data available were included in the age-adjusted models.

## Results

The characteristics of the study population are shown by location and level of education in table 1. Ghanaian participants in Europe had the highest proportion of individuals with higher levels of education (46.9% in men and 35.1% in women) compared with those in urban Ghana (31.5% in men and 11.5% in women) and rural Ghana (20.2% in men and 5.2% in women). A greater proportion of participants with a higher level of education were in the high occupational class in all locations. The proportion of obese participants (BMI≥30 kg/m^2^) was highest in those with a higher level of education among men in rural Ghana but not in Europe where obesity was high across all levels of education. In rural Ghana and Europe, the proportion of physically inactive participants was highest among those with a higher level of education in men and women. The majority of Ghanaians in Europe were first generation migrants (98.2 and 98.3% for men and women, respectively). The mean duration of residence in Europe for first generation Ghanaian migrants was 16.9 (SD=9.7) and 17.1 (SD=9.5) years for men and women, respectively.

**Table 1:** Baseline characteristics of study population by gender, location and level of education

Total number	Rural Ghana n=974			Urban Ghana n=1400			Europe n=2916		
	Elementary	Secondary	Higher	Elementary	Secondary	Higher	Elementary	Secondary	Higher
Men									
Number of participants, N	158	146	77	92	176	123	159	492	576
Mean age, years (SD)	47.7 (12.2)	46.0 (12.6)	43.3 (13.8)	47.9 (11.3)	46.7 (11.4)	45.5 (12.9)	50.0 (8.3)	47.7 (9.3)	46.3 (11.4)
Obesity BMI≥30 kg/m^2^, n (%)	0	1 (0.7)	3 (3.9)	3 (3.3)	11 (6.3)	11 (8.9)	28 (17.6)	93 (18.9)	100 (17.4)
High occupation class, n (%)	5 (3.3)	8 (5.7)	33 (44.0)	16 (17.8)	41 (23.8)	65 (54.6)	12 (10.3)	88 (22.5)	261 (55.8)
Low physical activity (%)	12 (7.6)	12 (8.3)	17 (22.1)	27 (30.3)	25 (14.4)	35 (28.7)	21 (15.5)	62 (20.1)	86 (24.9)
Total energy intake ≥3500 kcal/day, n (%)	19 (14.5)	12 (9.5)	11 (15.5)	2 (2.2)	6 (3.5)	7 (5.7)	15 (15.5)	62 (20.1)	86 (24.9)
Mean years since migration, (SD)*	–						17.6 (8.7)	16.9 (9.3)	16.8 (10.4)
Women									
Number of participants, N	397	165	31	522	371	116	476	621	592
Mean age, years (SD)	48.3 (12.3)	43.1 (11.9)	42.9 (13.9)	46.7 (11.1)	43.1 (10.6)	41.0 (12.0)	47.8 (7.8)	45.7 (9.2)	45.2 (10.8)
Obesity BMI≥30 kg/m^2^, n (%)	30 (7.6)	15 (9.2)	3 (9.7)	178 (34.1)	116 (31.3)	44 (37.9)	241 (50.8)	308 (49.8)	279 (47.4)
High occupation class, n (%)	36 (9.6)	17 (11.3)	18 (60.0)	172 (36.6)	123 (34.7)	70 (64.2)	26 (10.1)	139 (30.6)	332 (67.8)
Low physical activity, n (%)	90 (22.7)	38 (22.9)	10 (32.3)	244 (46.9)	115 (31.3)	47 (40.9)	89 (23.9)	161 (31.9)	154 (32.1)
Total energy intake ≥3500 kcal/day, n (%)	43 (12.4)	17 (11.9)	5 (18.5)	12 (2.3)	24 (6.7)	9 (7.8)	27 (9.0)	73 (19.5)	77 (23.8)
Mean years since migration, (SD)*							16.7 (8.3)	16.8 (9.8)	17.8 (10.2)

*Restricted to participants in Europe.

BMI, body mass index.

**Figure 1:**
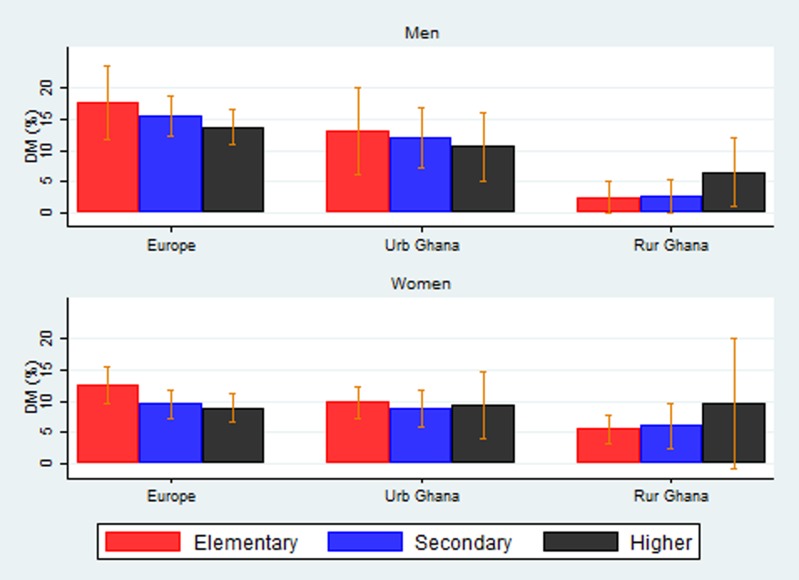
Prevalence of diabetes by education in men and women.

Figure 1 shows the age-standardised prevalence of diabetes by education in men and women by geographic location. The prevalence of diabetes decreased with increasing level of education in men and women in Europe and among men in urban Ghana. In rural Ghana, however, the prevalence of diabetes increased with increasing levels of education in men and women. There was no apparent pattern observed for women in urban Ghana.

**Figure 2:**
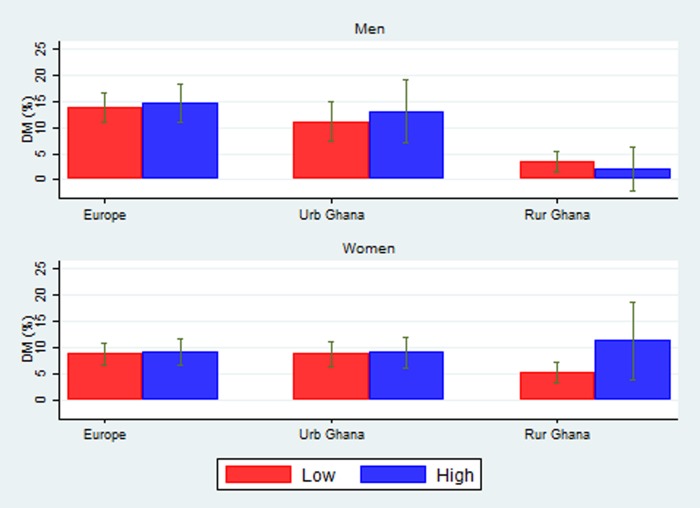
Prevalence of diabetes by occupational class in men and women.

Different patterns emerged for the prevalence of diabetes by occupational class as shown in figure 2. There was no observed difference in the prevalence of diabetes by occupational class in men and women in Europe and in women in urban Ghana. The prevalence of diabetes was higher in participants of a higher occupational class among men in urban Ghana and women in rural Ghana.

Table 2 shows the associations between the level of education and diabetes prevalence in men and women in rural Ghana, urban Ghana and Europe. In regression analyses, there were suggestions of a higher prevalence of diabetes in men and women with a higher level of education in rural Ghana. There were no significant associations between the level of education and the prevalence of diabetes in men and women in urban Ghana and in men in Europe. Women in Europe with a higher level of education, however, had a lower prevalence of diabetes (prevalence ratio (PR): 0.65; 95% CI 0.45 to 0.93). This association was not explained by BMI, physical activity and dietary intake.

**Table 2:** Association of level of education with diabetes in men and women

		Men			Women		
	Level of education	Distribution of diabetes (n/N)	Model 1	Model 2	Distribution of diabetes (n/N)	Model 1	Model 2
			PR (95% CI)	PR (95% CI)		PR (95% CI)	(PR (95% CI)
Rural Ghana	Elementary	4/158	1.00	1.00	22/397	1.00	1.00
	Secondary	4/146	0.98 (0.26 to 3.75)	0.70 (0.19 to 2.63)	10/165	1.31 (0.63 to 2.72)	1.34 (0.59 to 3.04)
	Higher	5/77	2.76 (0.76 to 10.08)	1.37 (0.40 to 4.79)	3/31	2.06 (0.75 to 5.67)	1.96 (0.82 to 4.64)
Urban Ghana	Elementary	12/92	1.00	1.00	51/522	1.00	1.00
	Secondary	21/176	1.15 (0.61 to 2.20)	1.46(0.75 to 2.88)	33/371	1.22 (0.79 to 1.90)	1.38 (0.89 to 2.15)
	Higher	13/123	1.03 (0.50 to 2.12)	1.11 (0.54 to 2.27)	11/116	1.28 (0.67 to 2.45)	1.37 (0.71 to 2.64)
Europe	Elementary	28/159	1.00	1.00	60/476	1.00	1.00
	Secondary	76/492	0.96 (0.64 to 1.45)	1.49 (0.79 to 2.79)	59/621	0.79 (0.56 to 1.11)	0.74 (0.46 to 1.20)
	Higher	79/576	0.91 (0.61 to 1.37)	1.33 (0.70 to 2.52)	53/592	**0.65 (0.45 to 0.93)**	0.68 (0.42 to 1.10)

Bold values are statistically significant.

Model 1- adjusted for age.

Model 2- Model 1 plus BMI, physical activity and dietary intake.

PR, prevalence ratio.

Table 3 shows the association between the prevalence of diabetes and the occupational class in men and women in the different locations. With the exception of women in rural Ghana in whom a higher occupational class was associated with a statistically significant prevalence of diabetes (PR: 2.92; 95% CI 1.41 to 6.06), no other associations were observed between the occupational class and diabetes prevalence in the other locations.

**Table 3:** Association of occupational class with diabetes in men and women

			Men			Women	
	Occupational class	Distribution of diabetes (n/N)	Model 1	Model 2	Distribution of diabetes (n/N)	Model 1	Model 2
			PR (95% CI)	PR (95% CI)		PR (95% CI)	(PR (95% CI)
Rural Ghana	Low	11/320	1.00	1.00	25/487	**1.00**	1.00
	High	1/46	0.70 (0.09 to 5.55)	0.73 (0.06 to 9.22)	8/71	**2.92 (1.41 to 6.06)**	2.28 (0.92 to 5.65)
Urban Ghana	Low	29/260	1.00	1.00	50/569	1.00	1.00
	High	16/122	0.92 (0.53 to 1.61)	0.85 (0.48 to 1.51)	33/365	1.15 (0.75 to 1.78)	1.16 (0.75 to 1.81)
Europe	Low	86/620	1.00	1.00	62/712	1.00	1.00
	High	54/364	1.06 (0.77 to 1.47)	1.34 (0.90 to 2.00)	47/509	0.91 (0.62 to 1.33)	0.95 (0.57 to 1.59)

Bold values are statistically significant.

Model 1- adjusted for age.

Model 2- Model 1 plus body mass index, physical activity and dietary intake.

PR, prevalence ratio.

## Discussion

There were differences in the patterns observed between the prevalence of diabetes and measures of SEP (educational level and occupational class) in Ghanaian men and women in the different locations in this study. In rural Ghana, the prevalence of diabetes was higher in men and women with a higher level of education. In contrast, diabetes prevalence was lower in Ghanaian men and women with a higher level of education in Europe. We did not observe a clear pattern in the association between the prevalence of diabetes and SEP in urban Ghana particularly among women. In regression analyses, with the exception of a lower prevalence of diabetes among Ghanaian women with the highest level of education in Europe and a higher prevalence of diabetes among women in rural Ghana with a higher occupational class, no other significant associations were observed. These relationships were not explained by BMI, physical activity and dietary intake. It is possible that differences in gender roles in the different settings may have contributed to these patterns among women.

The results of our study involving Ghanaians residing in different locations suggest differences in the social patterning of diabetes within different economic and social contexts as demonstrated in some previous studies. Whereas diabetes has been shown to be more prevalent in socioeconomically disadvantaged groups, in studies from high-income countries irrespective of the measure of SEP used,^1^ ^27^ ^28^ some studies from low-income countries have observed an increased prevalence of diabetes in higher SEP groups.^29^ The common explanation given for the observed inverse association in high-income countries is a higher risk of established risk factors of diabetes such as obesity, physical inactivity and increased consumption of unhealthy diets among people of lower SEP.^2^ ^30^ Access to healthcare services and health information may also contribute to socioeconomic differences in diabetes prevalence.^10^ Studies examining the association between SEP and the prevalence of diabetes in SSA are limited. The few studies available have generally shown a direct association with a higher prevalence or incidence of diabetes associated with higher SEP.^31^ ^32^ Higher SEP have been shown to be directly associated with obesity in most SSA studies, particularly in men which could possibly explain the observed direct association with diabetes.^33^ ^34^ People of higher SEP in SSA are more likely at this stage of their economic development to have higher prevalence of risk factors of diabetes such as obesity, because they are more likely to afford and therefore consume energy-dense foods and have lower levels of physical activity.^11^ The higher levels of physical inactivity in those with a higher level of education in rural Ghana in this study could possibly explain the direct association observed between SEP and diabetes in that location. Interestingly, among participants in Europe especially women, there were suggestions of an inverse association between SEP and the prevalence of diabetes. This inverse pattern was less consistent in Ghanaian men in Europe. Several studies in high-income and low-income countries have demonstrated a higher risk of diabetes in women with low SEP whereas the association in men has not always been consistent or statistically significant.^35–37^ We hypothesise that women with a higher level of education in Europe are more health conscious than men and possibly adapt lifestyles and behaviours that reduce their risk of developing of diabetes. The definite reasons for these sex-related differences, however, remain unclear and require further investigation. The patterns of the associations between diabetes prevalence and occupational class were less consistent. The reasons for this are uncertain but could be due to the classification of occupational class into just two categories (high and low) because of the low number of participants in some occupations. An education–occupation mismatch has been previously reported among migrant populations, where migrants with a high level of education are often engaged in unskilled jobs.^38^ Results from our study, however, indicate that the majority of participants with a higher level of education were of higher occupational class in all locations.

Previous studies have reported inconsistent patterns in the association between SEP and diabetes in migrant populations in Europe.^12^ ^14^ An earlier study which included South Asians and Europeans in the UK showed that whereas worse SEP was associated with a higher prevalence of glucose intolerance in Europeans, the patterns were less consistent and were more complex in South Asian men and women.^12^ The authors posited that the European pattern of inequalities was possibly established in different subgroups of South Asians at a different pace similar to the observed patterns in our study.^12^ Despite the inconsistent patterns often reported in the association between SEP and CVD risk factors in migrant populations, the general expectation is that a socioeconomic gradient will eventually emerge similar to that observed in the European host population.^39^ ^40^ The direct pattern of association between SEP and diabetes observed in rural Ghana and the inverse association observed in Europe, particularly among women in this study, is consistent with the ‘diffusion theory’ of the epidemic of ischaemic heart disease (IHD) mortality.^41^ This theory suggests that the rise in IHD started in higher socioeconomic groups in richer countries because they were the first to afford predisposing behaviours such as smoking and diets rich in saturated fats. The disease then spread to the lower socioeconomic groups when the epidemic started to decline because those in the higher socioeconomic groups were the first to adopt the behavioural changes required to reduce the risk of IHD.^41^ This could possibly explain the increased prevalence of diabetes in higher socioeconomic groups in rural Ghana but a decrease in prevalence in those of higher SEP in Europe.

There are some limitations to be considered in interpreting these data. Although we made every effort to increase the comparability of the data collected between locations, different recruitment strategies, different response rates and a different proportion of missing data in some covariates in the different locations may have affected the validity of our results. The assessment of the level of education and occupational class was based on identical questions that were modified to reflect the classifications used in each location. However, the majority of the participants in the RODAM study were first generation migrants who had completed most of their education in Ghana, therefore any differences in the classification of the exposure variables are unlikely to have significantly biased our results. In the absence of indicators of SEP such as income and wealth, the indicators used in this study (occupational class and education), may not have adequately captured all aspects of SEP that reflect the material resources available to participants in the different settings. Despite these limitations, the RODAM study provides important data on a relatively homogenous population of Ghanaians living in different geographic locations in Ghana and Europe.

## Conclusion

The results of this study demonstrated variations in the pattern of the association of diabetes and SEP by gender and by geographic location with a tendency for a direct association in rural Ghana and an inverse association in Europe. These findings highlighting the complex interaction of SEP and the development of diabetes may have important implications for diabetes prevention strategies in Ghanaians in different locations.

**What is already known on this subject?**

- Previous studies conducted in high-income countries have consistently shown lower socioeconomic position (SEP) to be associated with an increased prevalence of diabetes with the evidence suggesting a steeper gradient for women. The direction of the association between SEP and diabetes has, however, not been consistent in studies from low-income and middle-income countries and limited data is available on socioeconomic gradients in migrant populations in Europe.

**What this study adds?**

- This study, involving a relatively homogenous group of Ghanaians living in Ghana and Europe, demonstrated variations in the pattern of the association of diabetes and SEP by gender and by geographic location with a tendency for a direct association in rural Ghana (higher prevalence of diabetes in those with higher education) and an inverse association in Europe (higher prevalence of diabetes in those with lower education). These findings highlighting the complex interaction of SEP and the development of diabetes may have important implications for diabetes prevention strategies aimed at reducing health inequalities in Ghanaians in different locations.
